# Prostatic abscesses and severe sepsis due to methicillin-susceptible *Staphylococcus aureus*producing Panton-Valentine leukocidin

**DOI:** 10.1186/1471-2334-14-466

**Published:** 2014-08-27

**Authors:** Maria Dubos, Olivier Barraud, Anne-Laure Fedou, Fabien Fredon, Frédéric Laurent, Yannis Brakbi, Anne Cypierre, Bruno François

**Affiliations:** Intensive Care Unit, CHU Dupuytren, Limoges, France; Microbiology laboratory, CHU Dupuytren, Limoges, France; General surgery department, CHU Dupuytren, Limoges, France; National Reference Centre for Staphylococci, Hospices Civils de Lyon, Bron, France; Urology department, CHU Dupuytren, Limoges, France; Internal medicine department, CHU Dupuytren, Limoges, France; Service de Réanimation Polyvalente, Centre hospitalier universitaire Dupuytren, 2, avenue Martin Luther King, 87042 Limoges cedex, France

**Keywords:** Prostatic abscess, *Staphylococcus aureus*, Panton-valentine leukocidin, Severe sepsis

## Abstract

**Background:**

Prostatic abscesses are an uncommon disease usually caused by enterobacteria. They mostly occur in immunodeficient patients. It is thus extremely rare to have a Staphylococcal prostatic abscess in a young immunocompetent patient.

**Case presentation:**

A 20-year-old patient was treated with ofloxacin for a suspicion of prostatitis. An ultrasonography was performed because of persisting symptoms and showed acute urinary retention and prostatic abscesses. So the empirical antibiotic therapy was modified with ceftriaxone/amikacin. The disease worsened to severe sepsis and the patient was admitted in ICU. CT-scan and MRI confirmed three abscesses with perirectal infiltration and the bacteriological samples (abscesses and blood cultures) were positive to methicillin-susceptible *Staphylococcus aureus* producing Panton-Valentine leukocidine. The treatment was changed with fosfomycin/ofloxacin which resulted in a general improvement and the regression of the abscesses.

**Conclusion:**

*Staphyloccocus aureus* producing Panton-Valentine leukocidin are most commonly responsible for skin and soft tissue infections. To this day, no other case of prostatic abscess due to this strain but susceptible to methicillin has been described.

**Electronic supplementary material:**

The online version of this article (doi:10.1186/1471-2334-14-466) contains supplementary material, which is available to authorized users.

## Background

Prostatic abscesses have become uncommon and their incidence has dropped since the introduction of antibiotics [1]. They are mostly due to enterobacteria, especially *Escherichia coli*. Sometimes *Staphylococcus aureus* is involved [2] and most of the cases are due to methicillin-resistant strains in patients with immunodeficiency risk factors. These patients generally present with chronic or subacute infections which rarely progress into severe sepsis or septic shock. Some *Staphylococcus aureus* strains can produce Panton-Valentine leukocidin (PVL), this toxin being a virulence factor. We present here a case of prostatic abscesses due to methicillin-susceptible *Staphylococcus aureus* (MSSA) producing PVL in an immunocompetent patient.

## Case presentation

**Figure 1:**
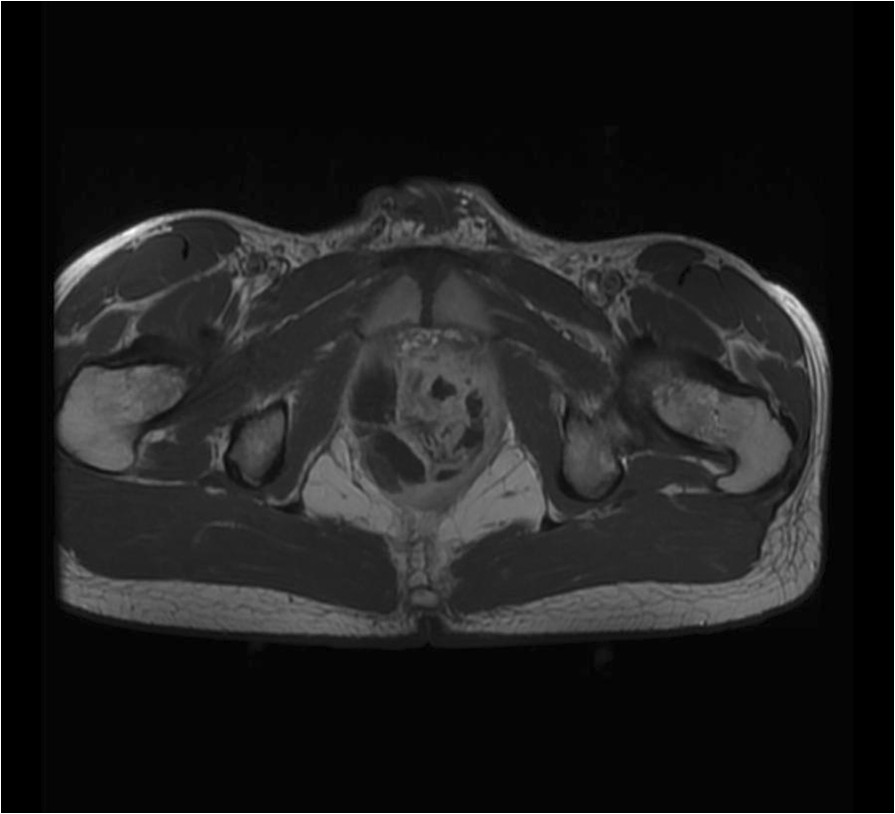
**Pelvic MRI at D0.** Pelvic MRI at D0 showing multiple intra-prostatic abscesses: the biggest ones are situated in the right lobe.

A 20-year-old patient was admitted in the hospital because of dysuria and burning during micturition. His medical history was unremarkable except for allergic asthma and recurrent furuncles for a year and a half with a negative HIV serology. Urine culture performed 1 day after symptoms appearance, showed a leukocyturia without bacteriuria so it did not require any treatment. Two days later, as the symptoms persisted, laboratory tests disclosed an inflammatory syndrome including a hyperleukocytosis (25,060 white blood cells/mm3) with a majority of neutrophilic leukocytes, a thrombocytosis and an increased level of C-Reactive protein (CRP) at 109 mg/L. Bladder ultrasound revealed a full bladder which resulted in the insertion of a suprapubic catheter and the initiation of oral ofloxacin. The patient was discharged home but presented again two days later with abdominal and pelvic pain. New laboratory tests showed persisting inflammatory syndrome and coagulation disorders (PT at 48%). The antibiotic therapy was replaced by ceftriaxone and amikacin. The prostate ultrasound revealed two abscesses and a transrectal needle aspiration collected 15 ml of purulent fluid with Gram positive cocci at gram stain.Six days later, condition worsened moving to severe sepsis with hyperthermia, chills and hypotension. The laboratory tests showed a worsening of the inflammatory syndrome (hyperleukocytosis = 43,000 white blood cells/mm3) and of the coagulation disorders (PT at 31%) and a hyperlactatemia at 2.90 mmol/l. Persistent perirectal abscesses were evidenced on the pelvic magnetic resonance imaging (MRI) (Figure 1). Ciprofloxacin was then added and the patient was transferred in the intensive care unit (ICU).

**Figure 2:**
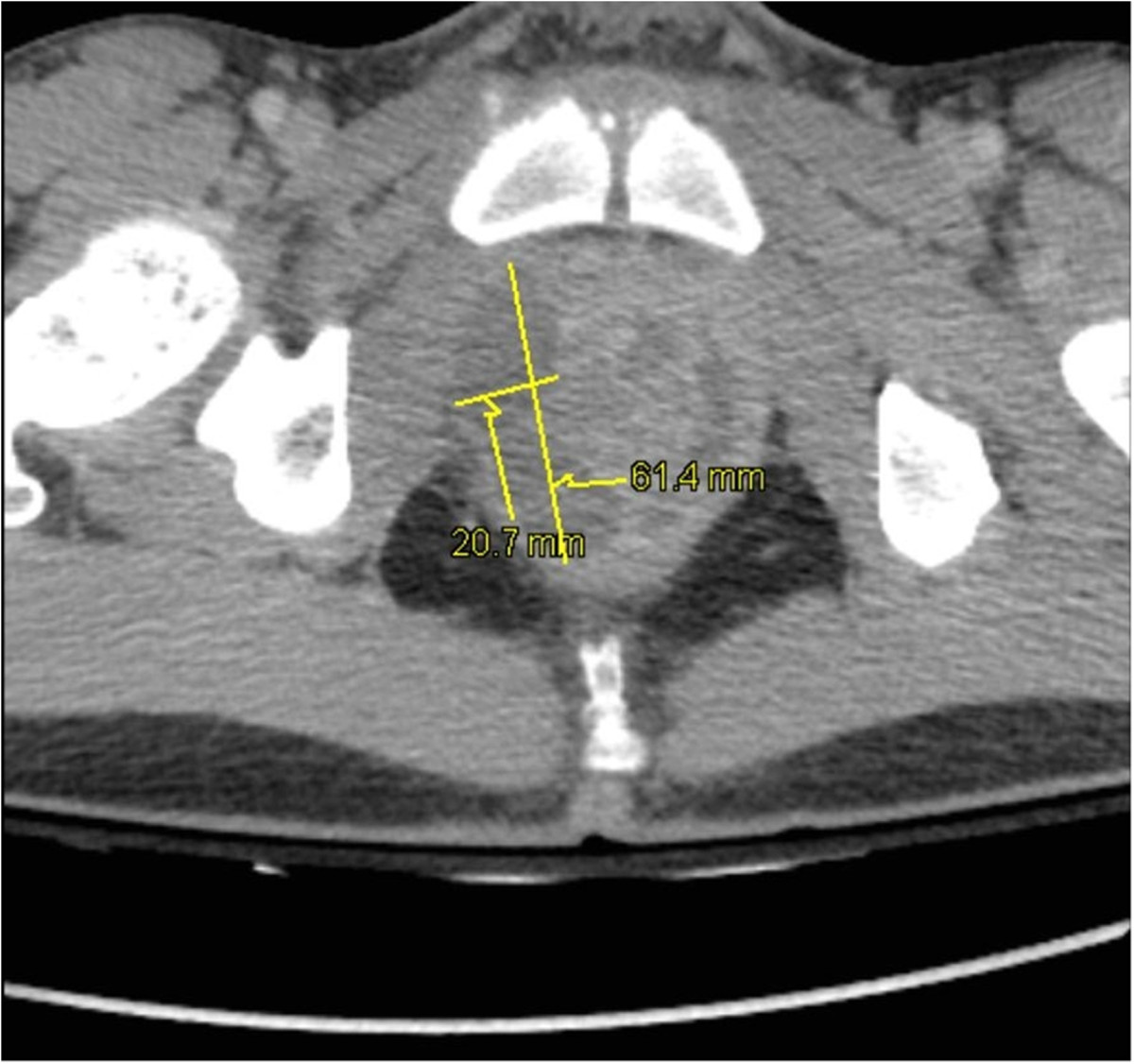
**Pelvic CT-scan.** Pelvic CT-scan showing prostatic hypertrophy and intra-prostatic abscesses: 20 × 15 × 33 mm and 64 × 21 × 26 mm in the right lobe and 38 × 10 × 30 mm in the left lobe.

**Figure 3:**
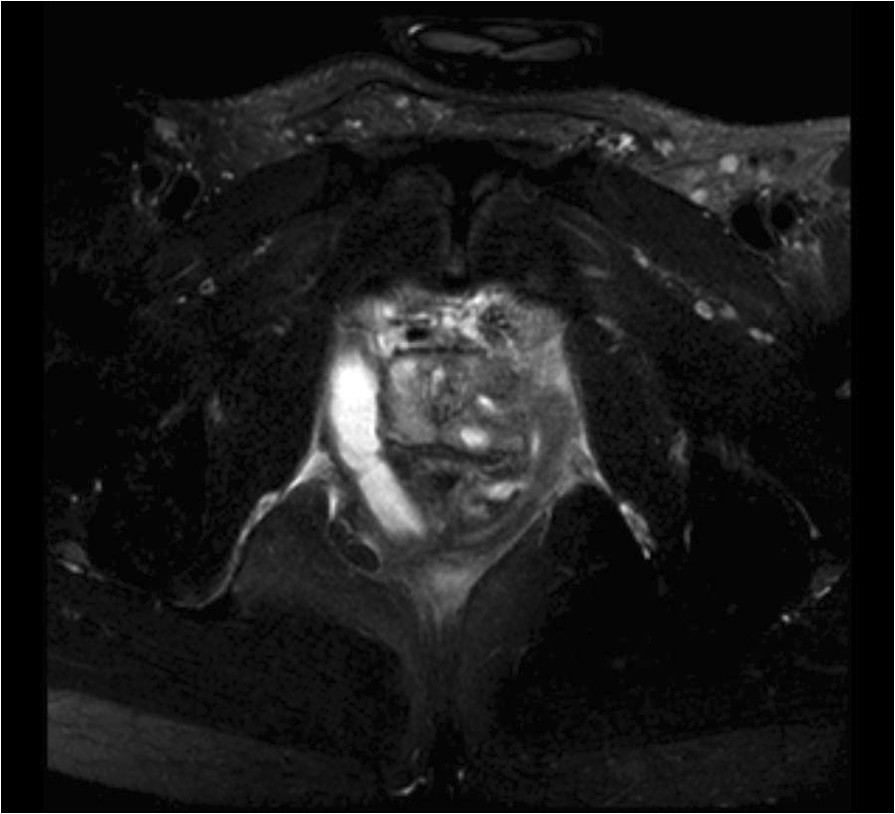
**Pelvic MRI at D16.** Control pelvic MRI at D16 showing regression of the abscesses in the left lobe and persistence in the right lobe.

At admission, the patient was hemodynamically unstable with a blood pressure at 98/52 mmHg requiring fluid resuscitation. The abdomen was painful. A CT-scan evidenced a clear prostatic hypertrophy with several abscesses (20 × 15 × 33 mm; 64 × 21 × 26 mm and 38 × 10 × 30 mm), a perianal infiltration and retroperitoneal, lateral-aortic and presacral enlarged nodes (Figure 2). Microbiological samples from prostatic abscesses and blood cultures were positive for MSSA producing PVL. Antimicrobial susceptibility tests showed that the strain was multisusceptible except for penicillin G, tetracycline and trimethoprim-sulfamethoxazole. Due to the atypical clinical presentation including multiple abscesses, we investigated by PCR the presence of genes encoding PVL [3] that was positive. Molecular characterization, performed by the French National Reference Centre for Staphylococci, using DNA microarray (Alere StaphyType DNA microarray; Alere Technologies GmbH, Jena, Germany), highlighted that the strain has an agr allele type 4, and was assigned to clonal complex CC121 [4] (MLST ST 121). Data confirmed that the strain harboured PVL genes but also genes encoding enterotoxins B, K and Q and the egc cluster. Treatment with fosfomycin and ofloxacin was started at admission (D0) without need for surgery.Apyrexia was obtained within 48 hours and the inflammatory syndrome improved. An echocardiography ruled out endocarditis. The antibiotic therapy lasted six weeks and was changed at D16 for oral rifampicin and ofloxacin. On the same day, control MRI showed a decline of the perirectal infiltration and a disappearance of the collection in the left side of the prostate (Figure 3). The suprapubic catheter was retrieved at D10 and patient’s micturition was normal again.

Evaluation for an oropharyngeal and nasal *Staphylococcus aureus* carriage realised by swab on day 17 was negative.

## Discussion and conclusions

Prostatic abscesses have become rare since the onset of antibiotics and are found in 0.5 to 2.5% of patients with a prostate inflammation [1]. They mostly occur in patients with local risk factors (urine retention, indwelling urethral catheter, chronic prostatitis…) or immunodeficiency risk factors [5].

The gold-standard for prostatic abscesses diagnostic is still transrectal ultrasonography [5, 6]. It allows a needle aspiration which is used to confirm the diagnostic and identify the microorganism involved [7]. CT-scan and MRI are useful to assess extension around the prostate or to look for remote focuses [8].

The most common etiologic agent is *Escherichia coli* but *Staphylococcus aureus* is often associated with prostatic abscesses [2]. Several cases of prostatic abscesses due to community-acquired methicillin-resistant *Staphylococcus aureus* (MRSA) have been reported in the literature [9–12]. In our case, the etiologic agent was MSSA producing PVL. Prevalence of *Staphylococcus aureus* producing PVL seems to have increased for several years: in 1995, Prevost *et al.* study reported 5% of strains in Western Europe [13] whereas in 2012, 36% of strains producing PVL were reported in the United-States [14]. This statement is particularly true for the USA. In Europe, it is unclear whether the prevalence has truly increased or if it is due to increased ascertainement. Moreover, in Europe, there is clonal heterogeneity [15]. In the USA, there is a strong association between strains producing PVL and MRSA [14]. In Europe, Australia and Africa, there is a high proportion of MSSA producing PVL [16–19]. In our case the strain producing PVL was susceptible to methicillin. MSSA strains producing PVL are often from the clonal complex CC121, ST 121 [20, 21].

The PVL is a synergohymenotropic toxin that acts through the association of two components F and S. It destroys cells by creating pores in the membrane [22] and is responsible for leukocyte and macrophage destruction and tissue necrosis. Its role in the pathogenesis and the spreading of infections is still unclear [23]. Strains producing PVL are usually linked with skin infections such as furuncles and abscesses, with necrotising pneumonia [23] and with bone and joint infections [24] that mostly occur in healthy children and young adults.

PVL may contribute to the infection severity and be a virulence factor. This could explain that our patient presented with severe sepsis. Studies conducted in animals showed that PVL led to the persistence of infection and made its local extension easier [25]. Other studies highlighted a higher frequency of hemoptysis, general signs (tachycardia, low blood pressure, polypnea and cyanosis) and deaths in pneumonia caused by *Staphylococcus aureus* producing PVL, which illustrates the virulence of the toxin [22]. In 2013, Shallcross *et al.* showed in a meta-analysis that infections were most likely to recur and surgery performed more often in skin and soft tissue infections caused by strains producing PVL [26]. Of note, the patient had a history of repetitive furuncles for a year and a half which could be linked with recurrence. To our knowledge, this is the first case of prostatic abscess due to *Staphylococcus aureus* producing PVL with the toxin possibly responsible for the severe sepsis.

Prostatic abscesses treatment usually includes an antibiotic therapy adapted to the microorganism and drainage of the abscess. Ultrasound-guided transrectal needle aspiration must be preferred to surgical drainage or transurethral prostatic resection as the needle path is shorter and the tolerance rate higher [27]. The antibiotic treatment aims at eradicating *Staphylococcus aureus* but it must also decrease the toxin effects. In 2008, a study evidenced the ability of linezolid, clindamycin and rifampicin to inhibit PVL production [28].

Prostatic abscesses must be searched for in all patients presenting with prostatitis symptoms associated with abdominal pain. A *Staphylococcus aureus* origin must be suspected if the disease worsens and develops to severe sepsis despite an adapted antibiotic therapy, especially in a young subject. Moreover, if this pathogen producing PVL is discovered, an antibiotic therapy including toxin inhibition must be started.

## Consent

Written informed consent was obtained from the patient for publication of this case report and of the images associated. A copy of the written consent is available for review by the Editor of this journal.

## Authors’ original submitted files for images

Below are the links to the authors’ original submitted files for images. Authors’ original file for figure 1 Authors’ original file for figure 2 Authors’ original file for figure 3

## Abbreviations

CRP: C-Reactive protein
HIV: Human immunodeficiency virus
ICU: Intensive care unit
MRI: Magnetic resonance imaging
MRSA: Methicillin-resistant *Staphylococcus aureus*
MSSA: Methicillin-susceptible *Staphylococcus aureus*
PCR: Polymerase chain reaction
PVL: Panton-Valentine leukocidin
PT: Prothrombin time.
