# Supplementary figures and images for: Prostatic abscesses and severe sepsis due to methicillin-susceptible Staphylococcus aureusproducing Panton-Valentine leukocidin
Source: BMC Infect Dis. 2014 Aug 27;14:466. doi: 10.1186/1471-2334-14-466 (PMC4156639; doi:10.1186/1471-2334-14-466)

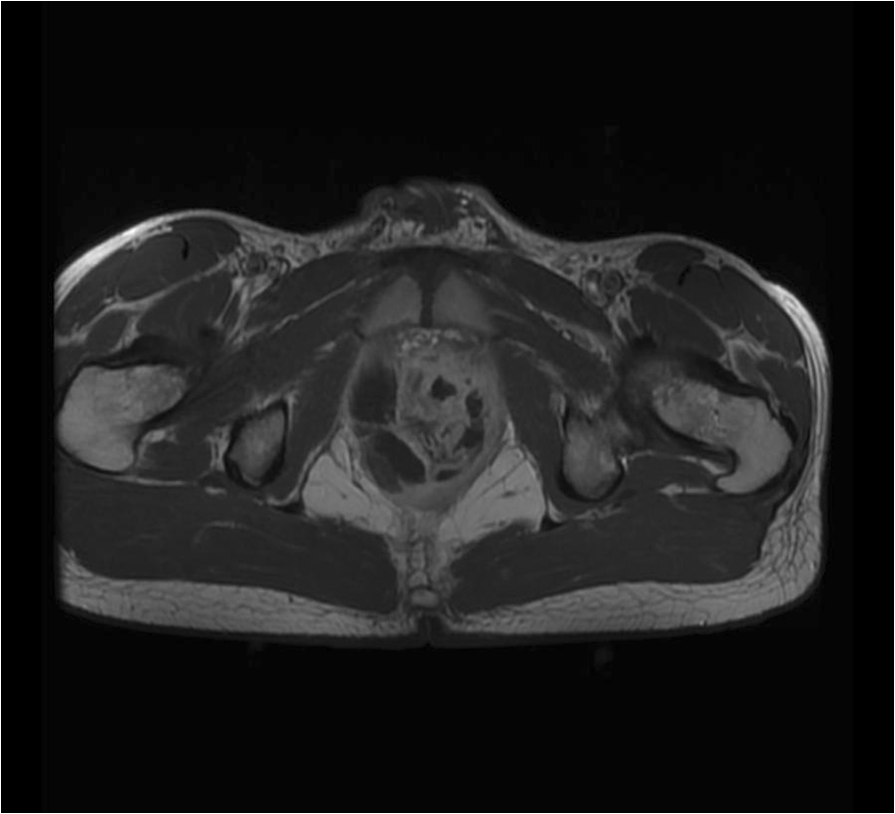

Supplement: Supplementary file 1 — Authors’ original file for figure 1 [file 12879_2014_3766_MOESM1_ESM.tif]

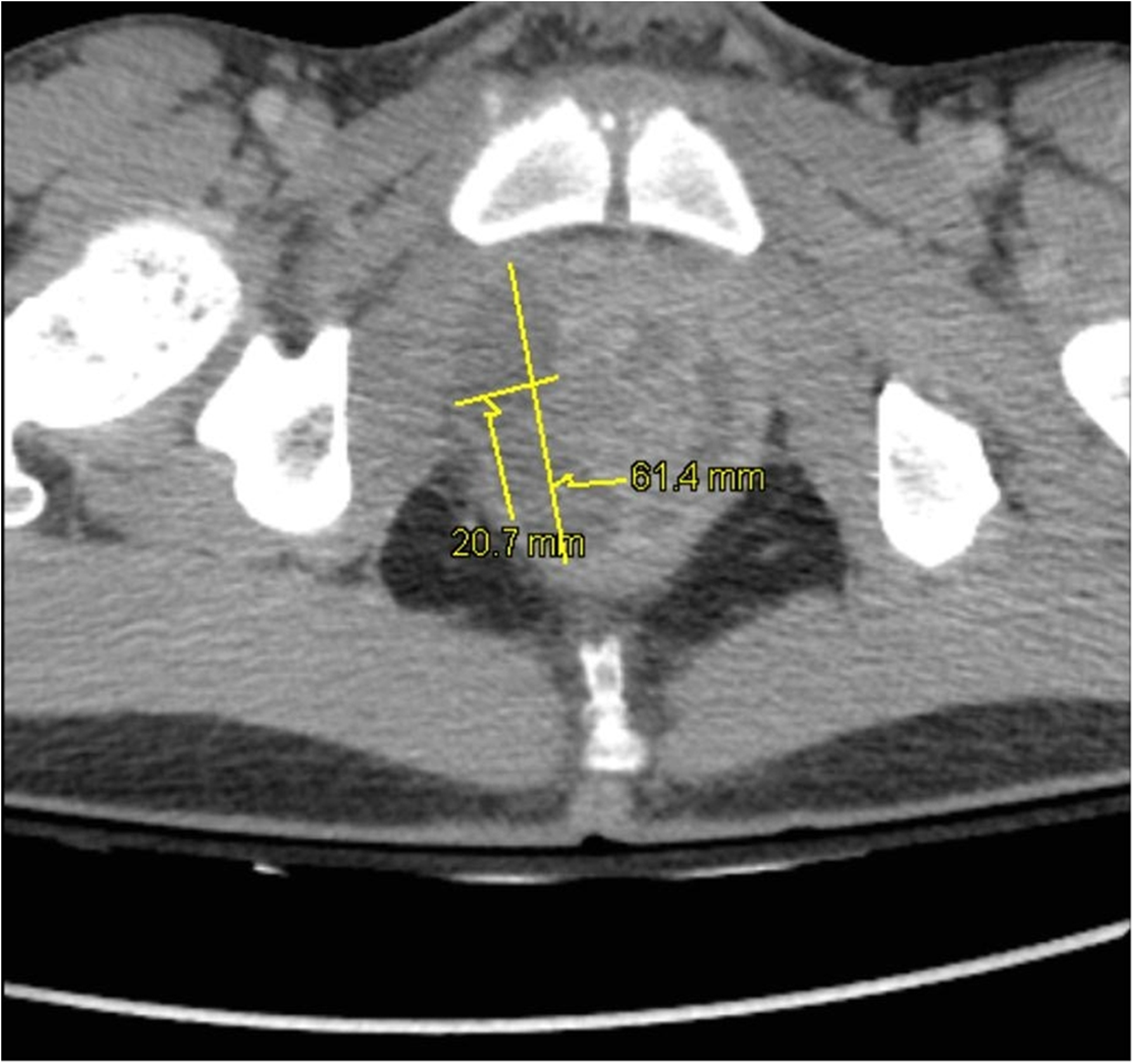

Supplement: Supplementary file 2 — Authors’ original file for figure 2 [file 12879_2014_3766_MOESM2_ESM.tif]

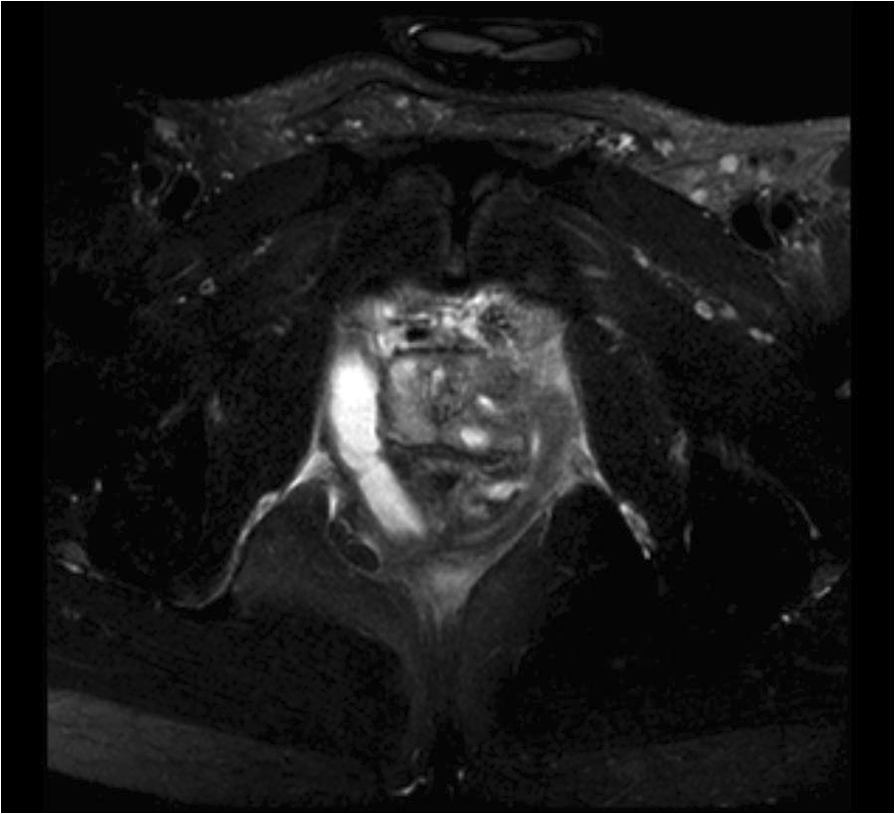

Supplement: Supplementary file 3 — Authors’ original file for figure 3 [file 12879_2014_3766_MOESM3_ESM.tif]
